# Unraveling the amygdala: A review of its anatomy and functions

**DOI:** 10.6026/9732063002001588

**Published:** 2024-11-30

**Authors:** K.S.V Angu Bala Ganesh, Payal Panda, Amit H. Makwana, Prarthana Kalerammana Gopalakrishna, Kandula Prameela Rani, Thirupathirao Vishnumukkala

**Affiliations:** 1 Department of Anatomy, Gujarat Adani Institute of Medical Sciences, Bhuj, Gujarat, India; 2 Department of Anatomy, C.U. Shah Medical College & Hospital, Surendranagar, Gujarat, India; 3 Department of Physiology, C.U. Shah Medical College & Hospital, Surendranagar, Gujarat, India; 4 Physiology discipline, Human Biology division, School of Medicine, IMU University, Kuala Lumpur, Malaysia; 5 Department of Microbiology, AIMST University, Kedah, Malaysia; 6 Anatomy discipline, Human Biology Division, School of Medicine, IMU University, Kuala Lumpur, Malaysia

**Keywords:** Amygdala, emotion, basal nucleus, anxiety

## Abstract

The amygdala is a complex cellular structure situated centrally in the brain, adjacent to the hippocampus. It is an integral part of the limbic system and is essential for the processing of emotional reactions. The amygdala possesses extensive connections to multiple brain regions, enabling it to acquire sensory information and affect responses. The amygdaloid complex has over ten nuclei located in the mid-temporal lobe. It is quite likely that the primary root of certain anxiety disorders in humans, such as posttraumatic stress disorder, is a dysfunction in the processing of information associated with fear. This article analyzes the anatomical and physiological foundations suggested to support amygdala function.

## Background:

**Figure 1:**
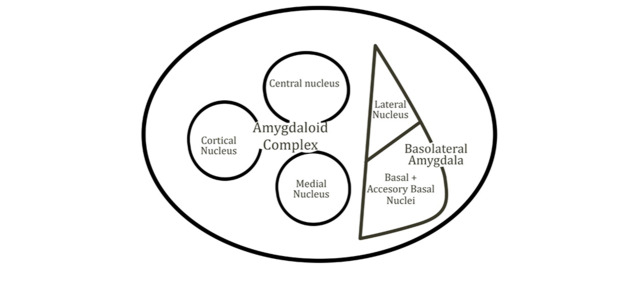
Amygdala and its subdivisions

The amygdala is a limbic structure comprised of a cluster of nuclei situated in the temporal lobe [1]. Despite its role, which is related with the processing of both unpleasant and rewarding environmental stimuli, it is crucial for the processing of emotions and for motivation [2]. The amygdaloid body is located within the depths of the temporal lobe and comprises 13 nuclei and their subdivisions. The nuclei of the amygdala are categorized into three groups: the basolateral nuclei, cortical-like nuclei and centromedial nuclei [3]. Additionally, there exists a set of nuclei that do not fall under these three categories, including intercalated cell masses, the amygdalohippocampal area and the substantia innominata, which collectively form the extended amygdala [4] (Figure 1).

## Basolateral nuclei:

The basolateral nuclei are separated into three primary nuclei: the lateral nucleus (LA), the basal nucleus (B), also known as the basolateral nucleus (BLA) and the auxiliary basal nucleus (AB) also known as basomedial nucleus. The LA is divided into three sections: dorsolateral, ventrolateral and medial. The BLA is divided into three main sections: rostral magnocellular, caudal intermediate and parvicellular. AB is divided into three sections: magnocellular, intermediate and parvicellular [4].

## Cortical nuclei:

Corticomedial nuclei are located on the surface of brain. Corticomedial nuclei include the accessory olfactory tract bed nucleus (BAOT), the peri amygdaloid cortex (PAC), the nucleus of the lateral olfactory tract (NLOT) and the anterior and posterior cortical nuclei (CoA and CoP). The PAC is divided into three sections: medial division, the sulcal division and the periamygdaloid cortex [5].

## Centromedial nuclei:

Central (CeA), medial (M) and bed nucleus of stria terminalis (BNST) are the three main components of the centro medial nuclei. The CeA is further subdivided into capsular (CeC), medial subdivision (CeM), intermediate (CeI) and lateral (CeL). The medial nucleus is divided into four parts: rostral, central (dorsal and ventral) and caudal [6].

## Other amygdaloid nuclei:

The other amygdala nuclei include the amygdalo-hippocampal area (AHA), the anterior amygdala region (AAA) and the intercalated nuclei. The AHA is made up of medial and lateral subdivisions [7].

## Extended amygdala:

The extended amygdala consists of the amygdala's central (CeA) and medial (M) nuclei, as well as the stria-terminalis and the sublenticular part of the substantia innominata [8].

The basolateral amygdala (BLA) is involved in both short- and long-term memories related to significant experiences, such as fear. Lesions of the BLA eliminate both short-term and longterm fear memory [9]. Moreover, in rodents, thalamus containing medial geniculate nucleus relays the auditory stimuli inputs and reaches the amygdala [10]. The cortico-medial group is made up of the amygdala cortical and medial nuclei, which receive information about the olfactory system from the pyriform cortex and the olfactory bulb [8]. The insula, hippocampus and rhinal cortices also provide important information to the amygdala [11]. Information from the subcortical region comes to numerous nuclei to the amygdala, comprising every neuromodulator system [12]. Amygdala outputs can be introduced in both cortical and subcortical regions of the brain. The structure of the central nucleus is engaged to several subcortical regions which are known to intervene in various physiologic, behavioural and autonomic and expressions of emotional state [13]. The basal and the accessory-basal nucleus are the principal projections of amygdala, which directed towards the cerebral cortex. These anatomical projections bring out the amygdala role in regulating various cognitive processes such as decision making, memory and attention [14].

## Functions of amygdala:

The amygdaloid nuclei of the limbic system are positioned ventrally, near to the piriform lobe. The hypothalamus, facial nerve nucleus, thalamic reticular nucleus, trigeminal nucleus, the olfactory bulb and cortex, ventral tegmental area (VTA), locus coeruleus, hippocampus and laterodorsally tegmental nucleus all are linked to the amygdaloid nuclei. The amygdala, a danger detector, uses sensory, cognitive and other information to identify potential threats and trigger autonomic responses, ensuring emotional significance in situations [15]. The prefrontal cortex's orbital region influences the amygdala, influencing emotional responses based on context or past experiences, such as fleeing from a snake encountered on a forest floor, but not in a zoo or pet shop. Furthermore, the amygdala is engaged in the process of implicit learning, particularly in situations that are emotionally laden (fear conditioning) [30]. Amygdala stimulation causes intense emotion, including fear and aggression. Damaging lesions in temporal lobe epilepsy are potential in stimulating the amygdala which results in panic attack at its extreme stage. Panic attacks are spontaneous, repeated incidents of terror which produce a sense of impending disaster without a clear cause. Scan images of positron emission topography (PET) revealed an elevated parahippocampalgyri blood flow, initiating from the right side parahippocampalgyrus. Decreased blood flow enhances the chances of occurrence of anxiety attacks [16].

## Basolateral nucleus of amygdala (Basal nucleus):

**Figure 2:**
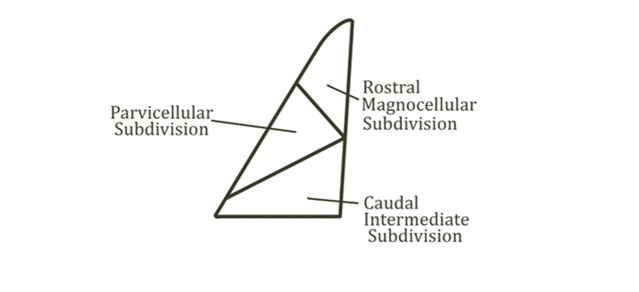
Basolateral nucleus and its subdivisions

The BLA is divided into three sections: rostral magnocellular, caudal intermediate and parvicellular. Average number of neurons in BLA: 115774, average volume of BLA: 1.20mm3 and average volume of rat brain: 994mm3 [17] (Figure 2).

## Connections and functions of basolateral nucleus of amygdala:

The basolateral nuclei of amygdala collect sensory input from the cortical association area, prefrontal cortex, thalamus and hippocampus through lateral nucleus. Basolateral nucleus processes this information and sends it to the central nucleus of amygdala through excitatory glutamatergic pathway along with relay of inhibitory GABAergic interneuron/intercalated neurons. The main output pathway of amygdala is central nucleus. Activation of inhibitory GABAergic neurons in the central nucleus leads to the development of autonomic and somatic signals of fear in the hypothalamus and brainstem. BLA also activates the cells in the BNST to the hypothalamus and brainstem structures for autonomic & somatic signs of anxiety [18]. Both central nucleus and BNST targets the same structures in the hypothalamus and brainstem but leads to a different activation pattern in regulation of hormonal and autonomic response. Central nucleus pathway triggers short term & result in sensation of fear and bed nucleus of stria-terminalis pathway leads to long-term activation & general signs of anxiety [19].

BLA has a dominant role in anxiety. Abnormal activity of BLA is observed in patients of anxiety [20]. After non-specific activation of glutamatergic BLA somata in animals, anxiogenic effects are elicited and are comparable with human data [21]. According to these studies, BLA has an adverse impact on social behavior. It has been established that the BLA plays an important role in reward behavior and that a lesion in the BLA decreases reward behavior, after BLA-nacinputactivation directs for reward seeking. The purpose of BLA neuronal activity is to decipher behavioral output rather than to identify conditioned stimuli. There is variety of neuronal reaction in the BLA, exact dissection on BLA channel is important in social, reward and anxiety related studies of BLA. It is necessary to identify the particular neuronal connection within BLA which is responsible for specific response, the basolateral amygdala stimulates the contextual and sensory inputs of fear and is included in fear memory and amygdala damage can cause problems with emotional reactions, memory processing and even decision-making [22].

## Cells in basolateral nucleus of amygdala:

Average number of neurons in BLA: 115774, average volume of BLA: 1.20mm3 and average volume of rat brain: 994mm3. BLA contains three types of cells: Glutamatergic pyramidal output neurons [Principal Neuron], GABAergic interneurons and Neuroglial cells [23]. Pyramidal projection neurons and non-pyramidal interneurons are two primary cell types present in basolateral nucleus of amygdala. Pyramidal-like projection neurons with spiky dendrites that use glutamate as an excitatory peptide are the main neurons in the basolateral amygdala. Non pyramidal neurons of the basolateral amygdala, on the other hand, are spineless interneurons that use GABA as an inhibitory neurotransmitter. The majority of glutamatergic neurons (80%) are primary or pyramidal and the GABAergic inhibitory interneurons accounting for the remaining 20%. Stress in any form affects pyramidal neurons in many ways especially affecting the morphology of the dendritic tree, the length of dendritic processes and the density of spines and synapses [24, 25].

## Amygdala and anxiety:

Fear, dread, unease and other unpleasant emotions are commonly referred to as anxiety. Condition anxiety is defined as an organism's influence throughout time and contexts, whereas state anxiety is defined as a response or adaptation to a current event. Anxiety is a negative-valenced emotion characterised by sustained hyper arousal in reaction to uncertainty; it is therefore future centered and aids in defence strategy or vulnerability assessments. An elevated plus maze, an open field and a light-dark box are the most common configurations used to investigate state anxiety. All of these methods exploit the rodent's desire for a cosy, dark or confined environment. Selective breeding results in high- and low-anxiety rodent strains and lines, which are commonly used in animal models. According to research in humans and animal models, the amygdala plays a distinct role in anxiety [26, 27].

## Kluver-Bucy syndrome:

Kluver-Bucy syndrome (KBS) is a rare neuropsychiatric condition caused by the abrasions which disturbs the hippocampus, bilateral temporal lobes of amygdala. The pathological characteristics are hyperorality, hypersexuality, hyper-metamorphosis, placidity, bulimia, visual agnosia and amnesia. The KBS clinical symptoms are produced by the destruction of the amygdala bilaterally and it is hardly observed in humans by their dysfunction in the anterior temporal lobe and its anatomical basis is still deliberated. However, KBS is caused by limbic networks instabilities of temporal portions which also modify emotional behaviour by affecting multiple cortical and subcortical circuits. Destructive lesions like ablation of the amygdala leads to an opposite effect from that of the damaging lesions of temporal lobe epilepsy. Damaging lesions of amygdala generate a placid calmness in humans and tameness in animals, which is featured as a flattening of the effect. Amygdaloid lesions can arise as a consequence of Urbach-Wiethe disorder in which calcium gets accumulated in the amygdala. The occurrence of the disorder in early life makes the patients unable to discriminate emotions through facial expressions. However, they would still be able to recognize faces. The region responsible for memory and face recognition is located in the multimodal association region of the infero-temporal cortex which shows the correlation between control from two different regions, which is emotion control in amygdala and perception control in the inferotemporal cortex, thereby making an intense emotion related memory [28, 29].

## Conclusion:

The amygdaloid complex's architecture, local connectivity, is now well understood. Fear complexity studies have taught the most about the amygdala and its afferent and efferent connections in this basic learning paradigm. Fear is a simple learning paradigm that clearly involves the amygdala. Physiological features of fear conditioning circuits will help explain brain mechanisms of emotional memory acquisition and storage. Disruptions to the amygdala can significantly affect emotional processing and behaviour, as seen in pathologies like Kluver-Bucy syndrome. Further research can deepen our understanding of neurological mechanisms underlying fear, anxiety disorders and therapeutic strategies.
